# Problematic Gaming and Sleep: A Systematic Review and Meta-Analysis

**DOI:** 10.3389/fpsyt.2021.675237

**Published:** 2021-06-07

**Authors:** Joakim H. Kristensen, Ståle Pallesen, Daniel L. King, Mari Hysing, Eilin K. Erevik

**Affiliations:** ^1^Department of Psychosocial Science, University of Bergen, Bergen, Norway; ^2^Norwegian Competence Centre for Gambling and Gaming Research, University of Bergen, Bergen, Norway; ^3^Optentia, Vaal Triangle Campus of the North-West University, Vanderbijlpark, South Africa; ^4^Norwegian Competence Center for Sleep Disorders, Haukeland University Hospital, Bergen, Norway; ^5^College of Education, Psychology, & Social Work, Flinders University, Adelaide, SA, Australia

**Keywords:** gaming addiction, gaming disorder, internet gaming disorder, sleep, sleep problems, insomnia, systematic review, meta-analysis

## Abstract

Problematic gaming has been linked to poor sleep outcomes; however, these associations have not yet been synthesized quantitatively. This review employed a meta-analysis to investigate the relationship between problematic gaming and sleep-related outcomes. A search of Medline, Embase, Web of Science, PsycINFO, and Google Scholar identified a total of 763 studies, including 34 studies (*n* = 51,901 participants) eligible for inclusion. Papers were included if available in any European language, addressed problematic gaming, contained original data, and provided sufficient data for calculation of effect sizes. Two researchers independently extracted data using pre-defined fields including quality assessment. Sleep-related outcomes were meta-analyzed for sleep parameters that were reported by 5 or more papers. Significant overall effects were found for sleep duration (*g* = −0.238, 95% *CI* = −0.364, −0.112), poor sleep quality (*OR* = 2.02, 95% *CI* = 1.47, 2.78), daytime sleepiness (*OR* = 1.57, 95% *CI* = 1.00, 2.46) and sleep problems (*OR* = 2.60, 95% *CI* = 1.94, 3.47). Between-study heterogeneity was detected for all meta-analyses. Subgroup analyses showed a higher inverse effect size for adolescent samples compared to adult or non-specific age samples in terms of sleep duration. For daytime sleepiness, a larger effect size was found for studies based on single-item sleep measures compared to multi-item sleep measures. For sleep problems, the subgroup analysis showed the opposite with a higher effect size for studies based on single-item sleep measures than multi-item sleep measures. Across all sleep parameters, problematic gamers consistently reported a more adverse sleep status than non-problematic gamers.

**Systematic Review Registration:** https://www.crd.york.ac.uk/PROSPERO/; record ID: CRD42020158955.

## Introduction

Gaming is a popular leisure activity worldwide (1). While gaming can be a beneficial activity for most individuals (2, 3), gaming becomes problematic for a minority of players who experience negative consequences of excessive gaming (4, 5). Such negative outcomes include depression, anxiety, loneliness, somatization, reduced quality of life, and poor academic achievement (5–10). Problematic gaming has largely been viewed as a behavioral addiction and classified based on the presence of addiction symptoms (i.e., salience, mood modification, tolerance, withdrawal, conflict, and relapse) (11). The inclusion of *Internet Gaming Disorder* (IGD) in the fifth edition of the Diagnostic and Statistical Manual of Mental Disorders (DSM-5) as a condition for further study (12) resulted in increased research on the condition. Further, problematic gaming received formal recognition as a mental/behavioral disorder by the World Health Organization (WHO) with the inclusion of *Gaming Disorder* (GD) in the 11th edition of the International Classification of Diseases [ICD-11; (13)]. Given the widespread definitions, lack of standardized measuring tools, and cutoff values to indicate problematic/pathological behavior, the prevalence rates of problematic gaming have varied significantly across studies ranging from 0.2 to 34% (14). Recent meta-analyses have estimated the average prevalence rate of problematic gaming to be 3.1–4.6% (15, 16). The empirical data have been more consistent showing that boys and men report more problems related to video gaming compared to girls and women and that problematic gaming is more common among younger than older subjects (10, 14, 17). It should be noted that the field of problematic gaming, including the diagnoses in the DSM-5 and ICD-11, involves several controversies, where both the conceptualizations and the measurements of problematic gaming have been criticized (18–20). For instance, some researchers have asserted that the etiology of problematic gaming is unknown and that the state of problematic gaming may reflect underlying difficulties (e.g., depression) rather than a problem in its own right (18). The controversies regarding problematic gaming substantiate the need for a better understanding of the relationship between problematic gaming and other difficulties.

As the levels of gaming have increased over the past years so have the prevalence rates of sleep problems (21). Furthermore, an increasing number of studies have found associations between problematic gaming and sleep problems (22–24). However, the strength of the relationship between problematic gaming and sleep problems has been inconsistent across studies and the directionality/causality remains unclear. Given the importance of sufficient sleep duration and quality for optimal functioning, subjective well-being, and good health, identifying potential determinants of sleep problems (e.g., problematic gaming) are of important scientific and practical interest. Moreover, as the negative outcomes associated with problematic gaming are similar to the negative outcomes observed in individuals with sleep problems (e.g., depression, reduced quality of life, poor academic achievement), insight into the sleep characteristics of problematic gamers could contribute to a better understanding of the phenomenon of problematic gaming.

There are a number of possible mechanisms by which gaming may influence sleep (25). As proposed by the media displacement hypothesis (26, 27), gaming could displace sleep directly as the individual chooses to engage in games over sleep, or indirectly by disregarding behaviors that are essential for good sleep hygiene (e.g., physical activity). For problematic gamers, the displacement may not be by choice but rather driven by an inability to stop playing. Arousal is another possible mechanism both due to social engagement, structural characteristics of the games (e.g., high event frequency), and the thrill related to winning or losing, which may interfere with sleep. The artificial blue-spectrum light emitted by screens projecting the visual gaming content may also directly enhance alertness and arousal (28), and suppresses nighttime melatonin secretion, important in regulating the sleep-wake cycle (29, 30). Followingly, late-night gaming may delay the sleep phase, making it difficult to fall asleep at needed or wanted times. Prolonged gaming may also negatively affect sleep by creating physical discomforts such as muscular pain and headache (31). Lastly, sleep may also be interfered by exposure to electromagnetic fields that are emitted by wireless gaming devices, which may alter the total sleep time, sleep efficiency, sleep architecture, as well as inhibit the secretion of melatonin (32–34).

Taken together, problematic gaming behavior may lead to shorter sleep duration, poorer sleep quality, delayed sleep phase, and problems initiating or maintaining sleep. However, the direction of the relationship could also be reversed. It is possible that individuals with insomnia and others experiencing sleep difficulties may use gaming to cope with sleeplessness and as such develop problematic gaming patterns. Also, there could be a bi-directional (reciprocal) relationship, meaning that problematic gaming could cause sleep problems which in turn could further fuel further problematic gaming and the other way around. Alternatively, it is possible that both problematic gaming and sleep problems are caused by common third variables. For example, both problematic gaming and insomnia have been shown to be strongly associated with depression (5, 35).

Given the growth in empirical evidence on the topic, there is a need for an updated review of the current knowledge on the sleep outcomes associated with problematic gaming. Moreover, there is to date no available meta-analysis quantifying the strength of the potential relationship between problematic gaming and sleep-related outcomes across studies. The following research questions were thus addressed: (1) What characterizes the studies conducted so far on the relationship between problematic gaming and sleep?, (2) what is the evidence for a causal relationship between problematic gambling and sleep? and (3) what is the strength of the association between problematic gaming and sleep problems? In an effort to consolidate the knowledge on the phenomenon the current study will use *problematic gaming* as an umbrella term for the wide range of related constructs used in the literature. Hence, problematic gaming is in this paper defined as a pattern of playing video games that could negatively influence physical and mental health and/or interfere with daily activities. It entails both online and offline gaming behavior and all types of digital gaming devices.

## Methods

### Search Strategy and Inclusion Criteria

A systematic literature review and meta-analysis were conducted in accordance with the PRISMA guidelines (36, 37). The meta-analysis was pre-registered at the PROSPERO International prospective register of systematic reviews (https://www.crd.york.ac.uk/PROSPERO/; record ID: CRD42020158955). A search strategy was developed by the authors and the literature searches were completed on January 11th, 2021, conducted using relevant keywords in Web of Science, Medline, Embase, PsycINFO, and Google Scholar electronic databases. Search words are displayed in Table 1. The search words were entered similarly in each database without any limits, filters, or use of specific MeSH-items. Additionally, the reference lists of relevant studies were examined. The search strategy yielded a total of 1,360 results, with the following result in each database: Web of Science (543 results), Medline (419 results), Embase (186 results), and PsycINFO (212 results). Due to a large number of results provided by Google Scholar (more than 18,800 hits), only the first 30 pages of results were reviewed; identifying five additional manuscripts.

**Table 1:** Search items.

	**Keywords/terms**	**Operand**		**Keywords/terms**
	**Problematic gaming**			**Sleep**
	“Problem^*^ gaming”			Sleep^*^
	“Gaming addiction”			Insomnia
	“Game addiction”			Circadian
	“Gaming disorder^*^”			“Morningness-eveningness”
	“Internet gaming”			“Delayed sleep”
	“Internet game”			“Social jet-lag”
	“Online gaming”			“Wake after sleep onset”
OR	“Online game^*^”	AND	OR	Snoring
	“Video gaming”			Hypersomnia
	“Video game^*^”			
	“Computer gaming”			
	“Computer game^*^”			
	“Excessive computer use”			
	“Risk^*^ gaming”			
	“Pathological gaming”			
	“Pathological internet”			

* *Truncation*.

For inclusion in the present review, the studies had to fulfill the following eligibility criteria: (1) The full manuscript was available in a European language (e.g., English, German, Spanish, French, Scandinavian languages, etc.), (2) The study addressed problematic gaming specifically; (3) The study contained original quantitative data on the relationship between problematic gaming and at least one sleep variable, and (4) the study reported estimates of, or sufficient data to calculate an effect size of, the strength of this relationship. No further restrictions in terms of publication time or design were implemented. In instances where studies reported insufficient data, the corresponding authors were contacted to provide the required data. Studies not reporting data on gaming specifically, but rather on problematic screen or internet behaviors were excluded.

### Data Extraction and Coding Procedures

The two main outcomes of interest were measures of problematic gaming and sleep-related outcomes assessed by self-report questionnaires, clinical interviews, or objective measurements. The data were extracted and coded using a coding schema encompassing the manuscript: Author(s), manuscript type, year published; study setting: Design, country, continent, year of data collection, ethical approval, conflict of interest; sample: Sample size and characteristics of participants, methodology: Collection method, instruments, instrument reliability, sleep parameter; and results: Type of estimate, effect size, and adjustment of confounding. Effect sizes were either extracted directly from the original publications or manually calculated. The extraction procedure was conducted by JHK and EKE who independently coded the studies. Discrepancies between the two reviewers were resolved by discussions. In studies categorizing subgroups of problematic involvement in games based on the endorsement of a number of symptoms (e.g., a polythetic scoring to indicate less severe “problematic gamer”/“at-risk” and a monothetic system to identify those who were “addicted”/“disordered”), the effect sizes of all/both groups were collapsed by calculating a mean effect size using a fixed effect-model ensuring that the control/contrast group were not counted more than once (38, 39). Also, one study reported data separately for sibling dyads (40). In this case, the effect sizes (and descriptive data) were collapsed for the dyads and corrected for the number of participants before entered into the meta-analysis. One study assessed problematic gaming using both the DSM-5 IGD and the ICD-11 GD criteria (41). In this instance, only the data based on the IGD criteria were included, for comparability with other studies in the present review. In cases where subscale data of the Pittsburgh Sleep Quality Index (PSQI) were reported, the global score estimate was coded as sleep quality while the subscale data were independently coded in terms of their respective sleep parameters. It should be noted that the associations between problematic gaming and sleep-related outcomes in the present meta-analysis are based on unadjusted relationships. Although some studies controlled for potential confounders, this was not conducted consistently across studies and the number and content of confounders adjusted for also varied. Thus, in order to extract comparable effect sizes for each study, we extracted unadjusted estimates and not parameters (e.g., standardized beta regression coefficients) correcting for confounders.

### Quality Assessment

Two reviewers (JHK and EKE) independently assessed the quality of the included studies using the Newcastle-Ottawa Quality Assessment Scale (NOS) adapted for cross-sectional studies (42, 43). The NOS assesses study quality in three categories; (1) the sample selection process; (2) comparability between groups; and (3) the ascertainment of the results. To determine the level of inter-rater reliability in the quality assessment procedure, Cohen's Kappa statistic was calculated and was found to be 0.667 (*p* < 0.001), indicating substantial agreement between the two reviewers. In the cases of disagreement between reviewers, consensus was sought through discussions.

### Meta-Analyses

Meta-analyses were conducted to calculate overall effect sizes for each sleep parameter separately. Based on previous reviews of the literature we expected a considerable variance in both terminology and the instruments used to measure problematic gaming as well as sleep parameters, as well as divergence in terms of study populations (44, 45). As such, the meta-analyses were a priori planned to use random-effects models for any sleep parameter that yielded five or more studies. If <5 specific studies could be identified for a given sleep parameter, the parameter was not included for meta-analysis as the results were judged to be too unstable and since random-effects models in such cases could yield an inappropriate estimation of the between-study variance. To quantify between-study variance, tests of heterogeneity were calculated using the *Q* and *I*^2^-statistics. The *Q*-statistic is a measure of the total observed study-to-study variation, and a significant *Q* indicates the presence of heterogeneity between the studies. The *I*^2^-statistic is a measure of percentage which quantifies the total amount of variability in a set of effect sizes that is a result of true differences between the studies. An *I*^2^ percentage of 25, 50, and 75 can roughly be interpreted as low, medium, and high levels of true heterogeneity, respectively (46).

Subgroup meta-analyses were conducted in an effort to explain the between-study variance. The impact of participant age was explored by creating a dichotomous moderator variable that separated studies of adolescents vs. adults. The weighted mean age was 15.1 and 23.6 for the two subgroups, respectively. In addition, a second dichotomous moderator variable separating studies that investigated sleep with a standardized multiple-item questionnaire and studies relying on single items (or own created) was created in order to assess the impact of this methodical discrepancy on the overall effect size. The subgroup analyses were based on mixed-effects models, using a random-effects model within subgroups (pooling within-group estimates of tau-squared) and a fixed-effect model across subgroups (38).

Publication bias was assessed for each sleep parameter by visual inspection of funnel plots and statistically by the Orwin's fail-safe *N* (47) and the Duval and Tweedie's trim and fill procedure (48, 49). The Orwin's fail-safe *N* is an estimate of how many missing studies with a specified effect are needed to bring the overall effect statistically to a pre-set level. If the *N* is low, there is concern that the whole overall effect is an artifact of publication bias as it is likely that some studies are missing due to publication bias. In the current study, *g* = 0.2 and *OR* = 1.2 were set as criteria for “trivial” effects, and *g* = 0.0 and *OR* = 1.0 were set as the mean of the missing studies. Lastly, Duval and Tweedie's trim and fill procedure was used to assess the magnitude of bias on the overall effect. It complements the funnel plot by imputing theoretically missing studies and adjust the overall effect size to the best estimate of an unbiased effect size. The extrapolated overall effect sizes are reported in terms of Hedges' *g* and odds ratios (*OR*). The Hedges' *g* is an estimation of the standardized mean difference between groups, which has the advantage over Cohen's *d* that it corrects for bias caused by small sample sizes. Hedges' *g* is interpreted in line with Cohen's (50) convention of small (0.2), medium (0.5), and large (0.8) effects. An *OR* of 2.0, 3.0, and 4.0 could be considered as small, moderate, and strong effects, respectively (51). All statistical analyses were conducted using Comprehensive Meta-Analysis, version 3.3.070 (Biostat, Inc., 2014).

## Results

### Study Selection

**Figure 1:**
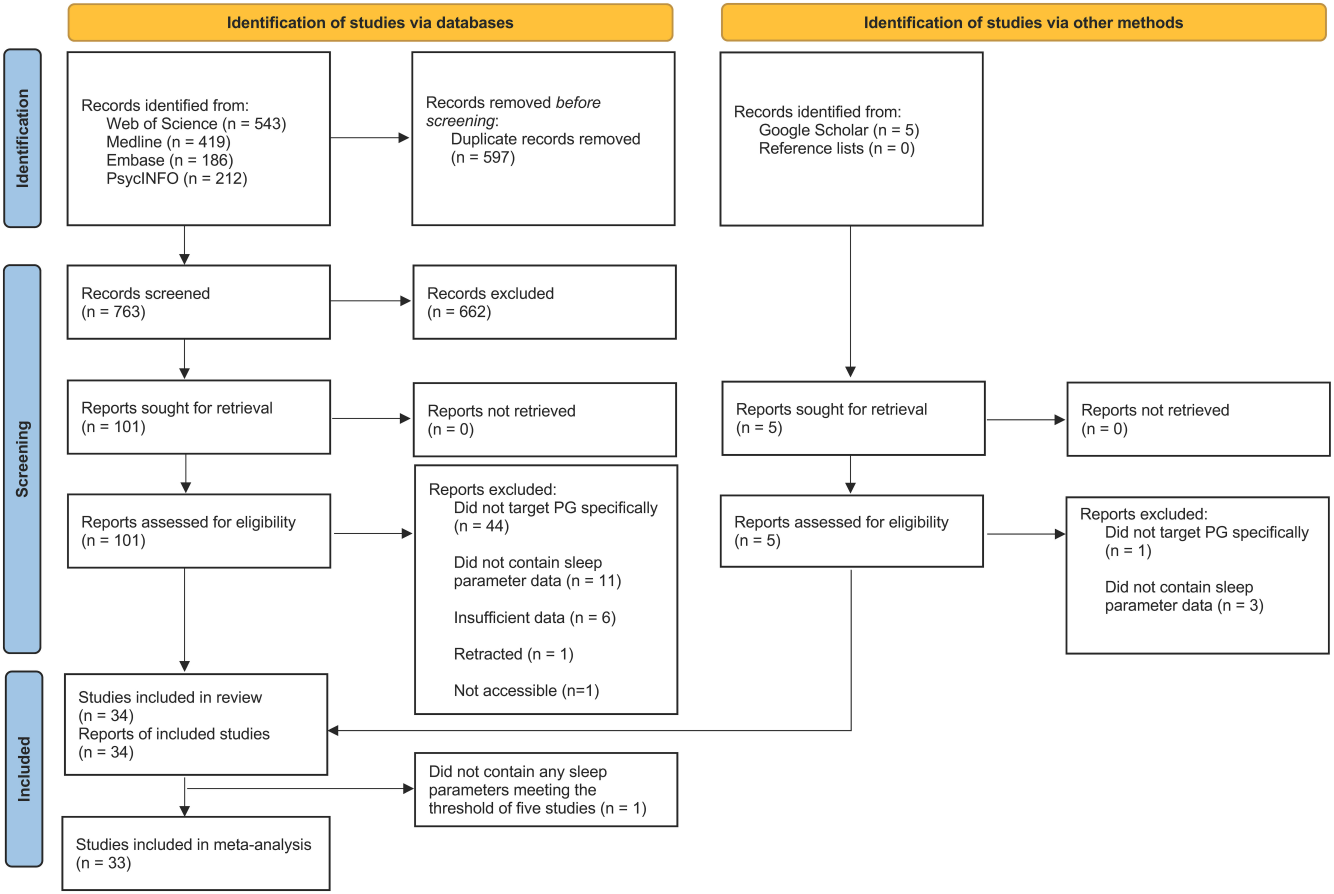
PRISMA 2020 flow diagram of the study screening and selection process.

Figure 1 presents a PRISMA flow diagram of the study screening and selection process. After removing duplicates (*n* = 597), the remaining (*n* = 763) titles and abstracts were independently screened by two reviewers (JHK and a research assistant), resulting in 101 studies for full-text eligibility evaluation. From this evaluation, 67 records were excluded resulting in 34 studies that met the eligibility criteria and were included in the review (see Figure 1 for reasons). From this pool, a total of 57 estimates (or results) on the relationship between problematic gaming and ten different sleep parameters were identified. Twelve studies reported sleep duration, 11 reported sleep quality, seven reported daytime sleepiness, 15 reported sleep problems, one reported sleep loss, one reported morningness-eveningness, one reported delayed sleep phase disorder, two reported bedtimes and wake-up times, and four studies reported other sleep-related outcomes.

### Descriptive Characteristics of the Included Studies

Table 2 summarizes the study characteristics and associations. Of all the 34 studies that met the eligibility criteria, 33 manuscripts were peer-reviewed research papers while one manuscript comprised an unpublished master-thesis (56). All manuscripts were published in English, except for one which was published in French (57). Regarding the study designs, one study used a longitudinal design (77), while the vast majority of studies (*n* = 32) were cross-sectional. Additionally, one study was a cohort study (65), but the sleep data were only collected at the first wave – making the data included in the present synthesis cross-sectional. The studies were published in the time frame of 2009–2021. The included studies yielded a summarized pool of 51,901 participants, with sample sizes ranging from 60 to 15,168 participants. The study populations came from 26 different countries with a majority originating from Europe. The participants' age ranged from 10 to 74 years with a weighted grand mean of 16.1 years. The proportion of girls/women ranged from 0 to 70.5%, with a weighted overall sex distribution of 30.7% girls/women for all the included studies. After merging subgroups of problematic gamers (e.g., “problematic”/“at-risk” and “addicted”/“disordered”), the included samples yielded a total of 5,625 individuals who were categorized as problematic gamers. Prevalence rates of problematic gaming varied widely in the samples ranging from 1.2 to 73.9% (weighted mean = 16.0%), while three studies had comparison groups of similar sample size (41, 71, 76). The majority of samples (*n* = 18) were recruited from primary- / high schools, while eight samples were recruited from college/universities (41, 53–55, 66, 75, 76, 80), three were recruited from gaming communities (24, 52, 74), one was recruited from two pediatric lipid and obesity treatment clinics (77), one was recruited through social media (61), one was recruited in “non-working contexts” [e.g., pubs, sports associations, recreational places; (59)], and two samples were recruited using random population sampling (56, 79).

**Table 2:** Study characteristics and associations.

**References**	**Country**	**Sample size (*n*)**	**Mean age (*SD*)/range**	**Sex (% females)**	**PG prevalence % (subgroups)^a^**	**PG instrument (α)**	**PG cutoff**	**Sleep instrument (α)**	**Sleep cutoff**	**Sleep parameter(s) and result(s)**		**NOS**
Achab et al. (52)	France	448	26.6 (7.1)/18–54	17.3	27.5	DAS (–)	≥ 3 of 7 criteria	Non-standardized questionnaire(–)	Continuous Yes/No Yes/No Yes/No	Sleep duration Sleep quality Daytimesleepiness Sleep deprivation	↓↓↑↑	6
Akçay and Akçay (53)	Turkey	935/892^c^	22.76 (2.2)/–	70.5	–	GASA-SF (0.81)	Continuous	PSQI (–)PSQI (–)ESS (–)Single item Single item	Continuous Continuous Continuous Continuous	Sleep quality Sleep duration Daytime sleepiness Later bedtime Later wake-up time	↓↑↑↓^*NS*^ ↑	5
Al Asqah et al. (54)	Saudi Arabia	228	21.15 (1.6)/18–25	35	28.1 (19.3/8.8)	IGDS9-SF (–)	≥2/≥5 of 9 criteria	Single item	Continuous	Sleep duration	↓	5
Al Gammal et al. (55)	Egypt	60	21.9 (2.7)/18–26	53.3	23.3 (18.3/5.0)	IGDS (–)	–	PSQI (–)	Continuous	Sleep quality	↑	4
Altintas et al. (24)	France	217	24.4 (6.98)/–	19.35	–	AIE-Q (0.84)	Continuous	PSQI (0.67)	High sleep quality/Low Sleep quality (HCA) and continuous	Sleep quality^d^	↓	6
Arnesen (56)	Norway	816	27.9 (7.36)/16–40	56.1	4.0	GASA (–)	≥4 of 7 items	BIS (0.82)	Continuous	Insomnia	↑	6
Bonnaire and Phan (57)	France	434	13.15 (0.5)/–	46.8	8.5	GASA (–)	≥4 of 7 items	Single item	Yes/No	Sleep problems	↓^*NS*^	5
Brunborg et al. (58)	Norway	1320	13.6 (0.32)/14–15	52.1	17.1 (12.9/4.2)	GASA (0.85)	2–3/≥4 core symptoms	Single items (0.60–0.70)	≥Once a week	Sleep problems Daytime sleepiness	↑↑	9
De Pasquale et al. (59)	Italy	566	22.74 (4.8)/18–35	42.76	5.3	IGDS9-SF (0.921)/DSM-5 IGD criteria (–)	Continuous/≥ 5 DSM-5 criteria	SCL-90R (–)	Continuous	Sleep disorders	↑	5
Fazeli et al. (60)	Iran	1512	15.51 (2.8)/13–18	43.6	–	IGDS9-SF (0.90)	Continuous	ISI (0.87)	Continuous	Insomnia	↑	8
Fernandes et al. (61)	India Indonesia Malaysia Mexico Philippines UK	185	21.59 (2.6)/–	65.76	–	GASA (0.89)	Continuous	Singe item	Continuous	Sleep quality	↓	3
Gonzalez-Valero et al. (62)	Spain	577	11.41 (0.5)/11–12	43.2	18.72 (17.5/1.2)	CESR (0.87)	≥26/≥39 test score	Single item	Continuous	Sleep duration	↓	4
Hawi et al. (23)	Lebanon	524	16.1 (1.0)/15–19	52.1	44.9 (35.7/9.2)	IGD−20 (0.91)	≥ 50 /≥ 71 test score	Single items	Continuous Sometimes or more	Sleep duration Woke up to continue playing	↓↑	7
Kim et al. (63)	South Korea	230	16.63 (1.0)/15–18	Males only	51.3	OGASA (0.93)	≥ 38 test score	Single item	> <6 hours of sleep	Sleep duration	↓	5
King et al. (64)	Australia	1287	14.9 (1.5)/12–18	50.4	–	PTU (–)	Continuous	SAMQ (–)	Continuous	Sleep duration^WD/WE^ Sleep onset latency^WD/WE^ Electronic media related sleep disruption	↓↑↑	5
Ko et al. (41)	Taiwan	207/138^c^	25.59 (3.8)/20–38	21.7	50^*^	Diagnostic interview based on DSM-IGD and ICD-GD criteria	IGD: ≥5 of 9 criteria GD: endorsement of 4, 5, 6, and 9^th^ criteria of IGD	Diagnostic interview	Yes/No Yes/No <1, 1-2, >3 a.m. <9 a.m., 9 a.m.-12 p.m., > 12 p.m.Yes/No Less than 4 h sleep 2 or more days a week	Insomnia Delayedsleep phase disorder Falling asleep at a later time Waking up at a later time Turning night into day Inadequate sleep time	↑↑↑↑↑↑	4
Lin et al. (65)	Iran	4,442	15.3 (1.6)/13–18	49.7	73.9 (47.9/26.0)	GAS(A) (0.89)	Latent class analysis (LCA)	PSQI (–)	Continuous	Sleep quality	↓	9
Lin et al. (40)	Iran	640	16.25 (2.6)/13–24	33.8	–	IGDS9-SF (–)	Continuous	ISI (–)	Continuous	Insomnia	↑	5
Liu et al. (66)	China	1,040	–	60.0	–	GASA (0.95)	Continuous	PSQI (0.64)	Continuous	Sleep quality	↓	4
Männikkö et al. (67)	Finland	293	18.7 (3.4)/13–24	49	8.2	GAS(A) (0.79)	≥ 4 of 7 items	Single item	Every week or more often	Sleep problems	↑	4
Männikkö et al. (68)	Finland	773	17.5 (4.4)/16–19	41.1	–	IGDT-10 (–)	Continuous	Single item	Continuous	Sleep duration	↓	5
Nakayama et al. (69)	Japan	814/549^c^	12.21 (0.4)/12-13	47.2	6.4	IGDT-10 (0.82)	≥3 test score	Single items	None–Always ≤ 21:59, 22:00-22:59, 23:00-23:59, 0:00-00-59. ≥ 1:00 h ≤ 5:59, 6:00-6:59, 7:00-7:59, 8:00-8:59, ≥ 9:00 h	Daytime sleepiness (during classes)Later bedtime^WD/HD^ Later wake-up time^WD/HD^	↑↑↑	6
Nogueira et al. (70)	Portugal	152	11.5 (–)/10–14	47	37.5 (33.6/3.9)	DSM-5 pathological gambling (–)	≥4/≥5 of 9 items	Single item PDSS (–)	Continuous Continuous	Sleep duration Daytime sleepiness	↑↑	2
Peracchia et al. (71)	Italia	300	14.76 (1.1)/–	62	50^*^	AICA-S (–)	4-6 h playing a day = “Hard gamers” <1 h playing a day = “Casual gamers”	PSQI (–)	Continuous	Sleep quality Daytime sleepiness Sleep efficiency	↑^*NS*^↓↑	4
Rehbein et al. (72)	Germany	15.168 /7761^b^	15.3 (0.7)/15–16	48.7/0 ^b^	6.7 (4.17/2.5)	KFN-CSAS-II (0.92)	≥ 35/≥ 42 test score	Single items	Continuous Always having problems falling asleep the week before	Sleep duration Sleep problems	↓↑	6
Rehbein et al. (73)	Germany	11.003	14.9 (0.7)/13–18	48.9	1.2	CSAS (0.93)	≥5 of 9 criteria	Single item	Continuous	Sleep problems	↑	6
Satghare et al. (74)	Singapore	1,085	23.7 (5.3)/13–40	44.5	–	IGDQ (0.73)	≥ 5 of 9 criteria	ISI (0.90)	≥ 10 test score	Insomnia	↑	6
Severo et al. (75)	Brazil	555	20.3 (5.4)/–	42.5	56.4 (18.2/38.2)	IGDS9-SF (–)	≥ 16/≥ 21 test score	PSQI (–)	Normal/Altered	Sleep quality	↓	7
Stockdale and Coyne (76)	USA	174	20.8 (2.2)/–^*^	15	50^*^	IGDS (–)	≥5 of 9 items	Neuro-QOL-SD-SF (–)	Continuous	Sleep problems	↑	7
Turel et al. (77)	USA	125	13.02 (2.2)/10–17	33	–	OVGA (0.87)	Continuous	*Fitbit*- actigraphy	Continuous	Sleep duration	↓	5
Vollmer et al. (78)	Turkey	471	12.89 (1.1)/11–16	39.7	–	CGA (0.92)	Continuous	CSM (–)	Continuous	Eveningness	↑	7
Wenzel et al. (79)	Norway	3405	–^*^/16–74	–^*^	1.4	Single engagement item	≥4 h playing a day	–	–	Sleep problems	↑	5
Wong et al. (80)	Hongkong/China	300	20.89 (1.5)/–	59.33	–	IGDS9-SF (0.91)	Continuous	PSQI (0.83)	Continuous	Sleep quality	↓	6
Yu et al. (81)	China	1,066	12.67 (–)/12–13	43.5	13.6	DSM-5 IGD criteria (0.75)	Continuous	ISI (0.84)	Continuous	Insomnia	↑	7

*↓, inverse association with problematic gaming; ↑, positive association with problematic gaming; –, not reported in the manuscript*;

* *, balanced comparison groups*;

NS *, not significant; NOS, Newcastle-Ottawa quality assessment scale score; α, Cronbach's alpha*;

a *, collapsed subgroups of problem gamers, subgroups in brackets (e.g., problem/disordered)*;

b *, only boys were included in the analysis*;

c *, included in the analysis*;

d *, all PSQI-subscales are reported*;

WD/WE *, Weekdays and weekends*,

WD/HD *, Weekdays and holiday*.

### Problematic Gaming Assessment

Most studies (*n* = 33) assessed problematic gaming using self-report questionnaires, while one collected the data through diagnostic interviews (41). Seventeen studies employed self-report instruments based on the DSM-5's IGD criteria, while seven studies used the Gaming Addiction Scale for Adolescents [GASA; (56–58, 61, 65–67)], one study adapted the criteria of pathological gambling (70), four studies adapted the DSM-IV-TR or the ICD-10 substance dependence criteria (24, 52, 71, 72), whereas four studies used instruments that were based on general addiction symptoms (62, 63, 77, 78). In addition, one study assessed problematic gaming exclusively based on time spent playing, with four hours or more playing per day defined as “excessive gaming” (79). Thirteen studies employed a continuous measure of gaming problems, while the majority (*n* = 21) reported comparisons of problematic gamers and non-problematic gamers; mostly identified using polythetic cutoff values (see Table 2).

### Sleep Assessment

The majority of studies (*n* = 32) investigated sleep using self-report questionnaires, while one study objectively measured sleep duration by employing *Fitbit*-actigraphy to register rest/activity cycles (77). Regarding self-report measures, 17 studies employed standardized sleep measurements where the most used instrument was the PSQI (*n* = 8), while five studies employed assessments for insomnia (40, 56, 60, 74, 81), and two studies assessed for daytime sleepiness (53, 70). The remaining studies assessed sleep-related outcomes using single/own-created items (see Table 2). In addition, one study collected sleep data through diagnostic interviews assessing for insomnia and delayed sleep phase (41).

### Quality Assessment

The quality scores of the single included studies ranged from two to nine stars, with a mean quality score of 5.5 and a standard deviation of 1.5 (see Table 3).

**Table 3:** Results from the Newcastle-Ottawa quality assessment.

	**Selection**				**Comparison**	**Outcome**	
**References**	**Representativeness (Max:⋆)**	**Sample size (Max:⋆)**	**Non-respondents (Max:⋆)**	**Ascertainment of the exposure (Max:⋆⋆)**	**Comparable outcome groups/Controlled for confounding factors (Max:⋆⋆)**	**Assessment of outcome (Max:⋆⋆)**	**Statistical test (Max:⋆)**
Achab et al. (52)	–	⋆	–	⋆	⋆⋆	⋆	⋆
Akçay and Akçay (53)	⋆	⋆	–	⋆⋆	–	⋆	–
Al Asqah et al. (54)	⋆	⋆	–	⋆⋆	–	⋆	–
Al Gammal et al. (55)	–	–	–	⋆⋆	–	⋆	–
Altintas et al. (24)	–	–	⋆	⋆⋆	⋆⋆	⋆	⋆
Arnesen (56)	⋆	⋆	⋆	⋆⋆	–	⋆	–
Bonnaire and Phan (57)	–	⋆	⋆	⋆⋆	–	⋆	–
Brunborg et al. (58)	⋆	⋆	⋆	⋆⋆	⋆⋆	⋆	⋆
De Pasquale et al. (59)	–	⋆	–	⋆⋆	⋆	⋆	–
Fazeli et al. (60)	⋆	⋆	–	⋆⋆	⋆⋆	⋆	⋆
Fernandes et al. (61)	–	–	–	⋆⋆	–	⋆	–
Gonzalez-Valero et al. (62)	–	⋆	–	⋆⋆	–	⋆	–
Hawi et al. (23)	⋆	⋆	–	⋆⋆	⋆⋆	⋆	–
Kim et al. (63)	⋆	⋆	–	⋆⋆	–	⋆	–
King et al. (64)	⋆	⋆	–	⋆⋆	–	⋆	–
Ko et al. (41)	–	⋆	–	⋆⋆	⋆	⋆	–
Lin et al. (65)	⋆	⋆	⋆	⋆⋆	⋆⋆	⋆	⋆
Lin et al. (40)	⋆	⋆	–	⋆⋆	–	⋆	–
Liu et al. (66)	–	⋆	–	⋆⋆	–	⋆	–
Männikkö et al. (67)	⋆	–	–	⋆⋆	–	⋆	–
Männikkö et al. (68)	⋆	⋆	–	⋆⋆	–	⋆	–
Nakayama et al. (69)	⋆	⋆	⋆	⋆⋆	–	⋆	–
Nogueira et al. (70)	–	–	–	⋆	–	⋆	–
Peracchia et al. (71)	–	⋆	–	⋆⋆	–	⋆	–
Rehbein et al. (72)	⋆	⋆	⋆	⋆⋆	–	⋆	–
Rehbein et al. (73)	⋆	⋆	⋆	⋆⋆	–	⋆	–
Satghare et al. (74)	⋆	⋆	–	⋆⋆	–	⋆	⋆
Severo et al. (75)	–	⋆	–	⋆⋆	⋆⋆	⋆	⋆
Stockdale and Coyne (76)	⋆	⋆	–	⋆⋆	⋆⋆	⋆	–
Turel et al. (77)	–	⋆	–	⋆⋆	–	⋆⋆	–
Vollmer et al. (78)	–	⋆	–	⋆⋆	⋆⋆	⋆	⋆
Wenzel et al. (79)	⋆	⋆	⋆	⋆	–	⋆	–
Wong et al. (80)	–	–	–	⋆⋆	⋆⋆	⋆	⋆
Yu et al. (81)	⋆	⋆	–	⋆	⋆⋆	⋆	⋆

### Meta-Analyses

Of all 34 studies and sleep parameters reviewed, sleep duration, sleep quality, daytime sleepiness, and sleep problems comprised the parameters that reached the threshold of five different effect sizes. A total of 33 studies were included in the quantitative meta-analysis.

#### Sleep Duration

**Figure 2:**
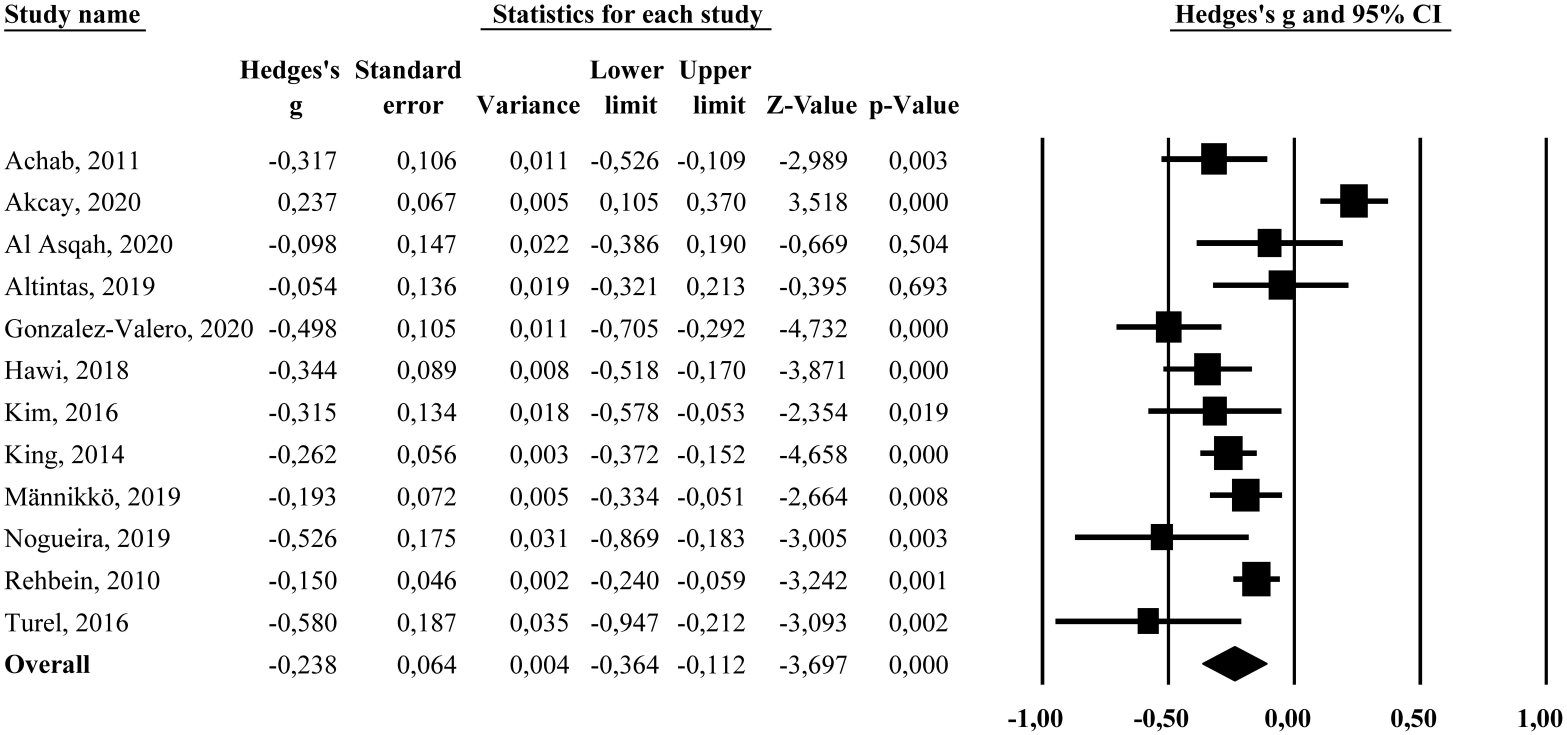
Forest plot showing the association (Hedges's *g*) between problematic gaming and sleep duration.

The forest plot regarding sleep duration is displayed in Figure 2. The results from the random-effects model showed a significant overall inverse association between problematic gaming and sleep duration (*g* = −0.238, 95% *CI* [−0.364, −0.112]), suggesting that problematic gamers report shorter sleep duration than non-problematic gamers. An additional random-effects model was performed for the studies providing data on sleep duration in terms of hours and minutes (*k* = 6). This amounted to a difference between conditions of −20.79 min (95%, *CI* = [−27.31, −14.30, min.]). The *Q*-statistic was significant (*Q* = 65.7, *df* = 11, *p* < 0.001), indicating heterogeneity between the studies. The *I*^2−^statistic showed a high percentage of true between-study variance (*I*^2^ = 83.3%). A subgroup analysis comparing studies targeting adolescent populations (*k* = 8, weighted mean age = 15.2) and studies targeting adult or non-specific age populations (*k* = 4, weighted mean age = 23.7), showed that the former subgroup had a higher overall effect size (*g* = −0.323, 95% *CI* [−0.450, −0.195]) compared to the latter subgroup (*g* = −0.037, 95% *CI* [−0.222, 0.148]). The difference between the subgroups was significant (*Q*_bet_ = 6.23, *df* = 1, *p* = 0.013). There was significant heterogeneity within the adolescent (*Q* = 17.86, *df* = 7, *p* < 0.05, *I*^2^ = 60.8%) and adult or non-specific subgroup (*Q* = 21.24, *df* = 3, *p* < 0.001, *I*^2^ = 85.9%). Concerning publication bias, the funnel plot was somewhat asymmetric. The trim and fill procedure suggested that two studies were missing and when imputing these the overall effect size was adjusted to *g* = −0.196, 95% *CI* [−0.317, −0.074].

#### Sleep Quality

**Figure 3:**
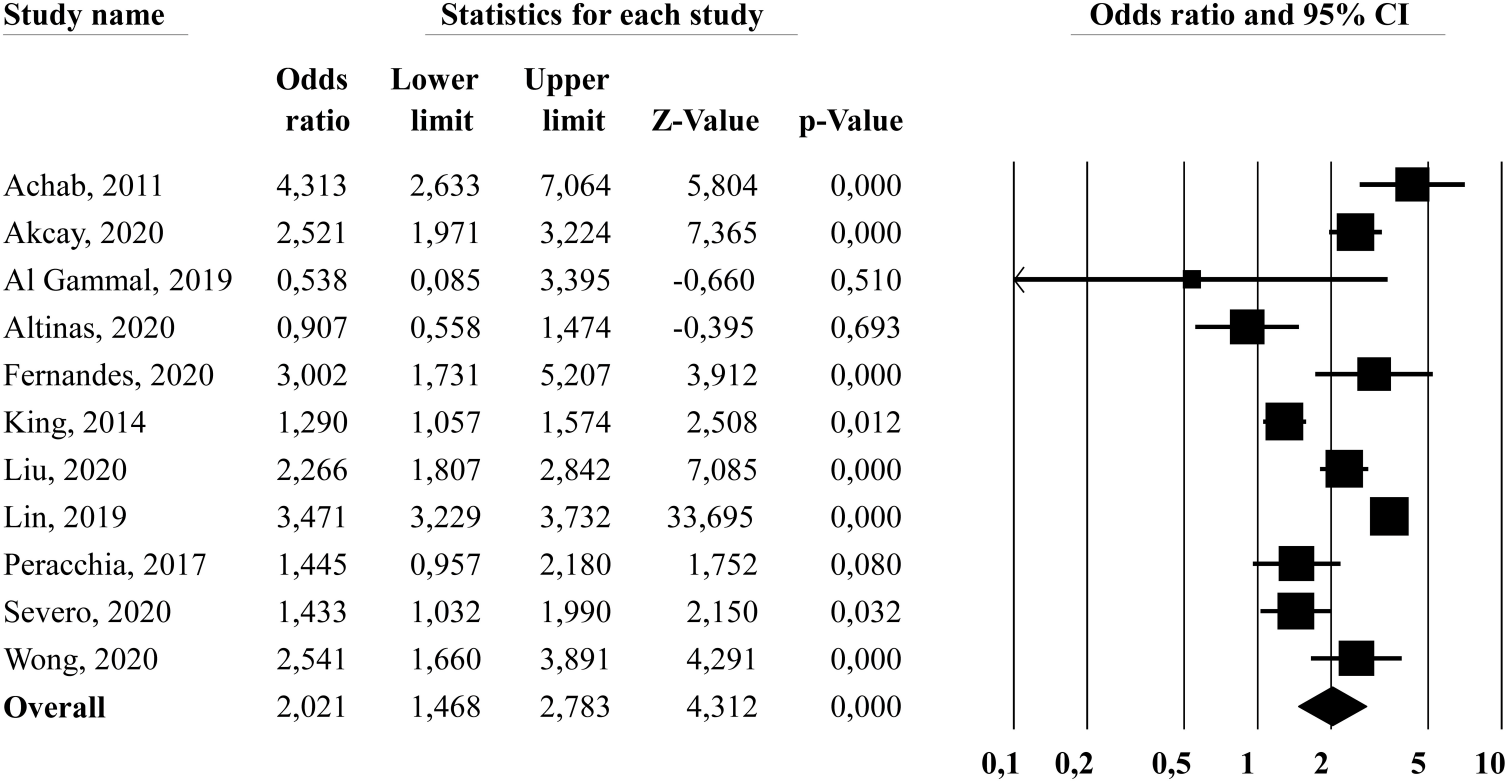
Forest plot showing the association (odds ratio) between problematic gaming and sleep quality.

Figure 3 displays the forest plot for sleep quality. The random-effects model showed that problematic gamers had increased odds of reporting poorer sleep quality compared to non-problematic gamers (*OR* = 2.02, 95% *CI* [1.47, 2.78]). The *Q*-statistic was significant (*Q* = 149.06, *df* = 10, *p* < 0.001), and the *I*^2−^statistic showed a high percentage of true between-study variance (*I*^2^ = 93.3%). The subgroup analysis comparing studies targeting adolescent populations (*k* = 3, weighted mean age = 15.2) and studies targeting adult/non-specific age populations (*k* = 8, weighted mean age = 22.9) did not yield any significant differences between subgroups (*Q*_bet_ = 0.05, *df* = 1, *p* = 0.819). A second subgroup analysis comparing studies that used a single-item sleep assessment (*k* = 2) and studies using multi-item sleep questionnaires (*k* = 9) was conducted. The difference between the subgroups was not significant (*Q*_bet_ = 2.56, *df* = 1, *p* = 0.109). The funnel plot was slightly asymmetric, and the trim and fill procedure suggested that one study was missing, adjusting the overall effect to *OR* = 2.09, 95% *CI* (1.52, 2.86). The Orwin's fail-safe *N* showed that 52 missing studies with zero effect are needed to reduce the overall effect to the trivial effect criterion (*OR* = 1.2).

#### Daytime Sleepiness

**Figure 4:**
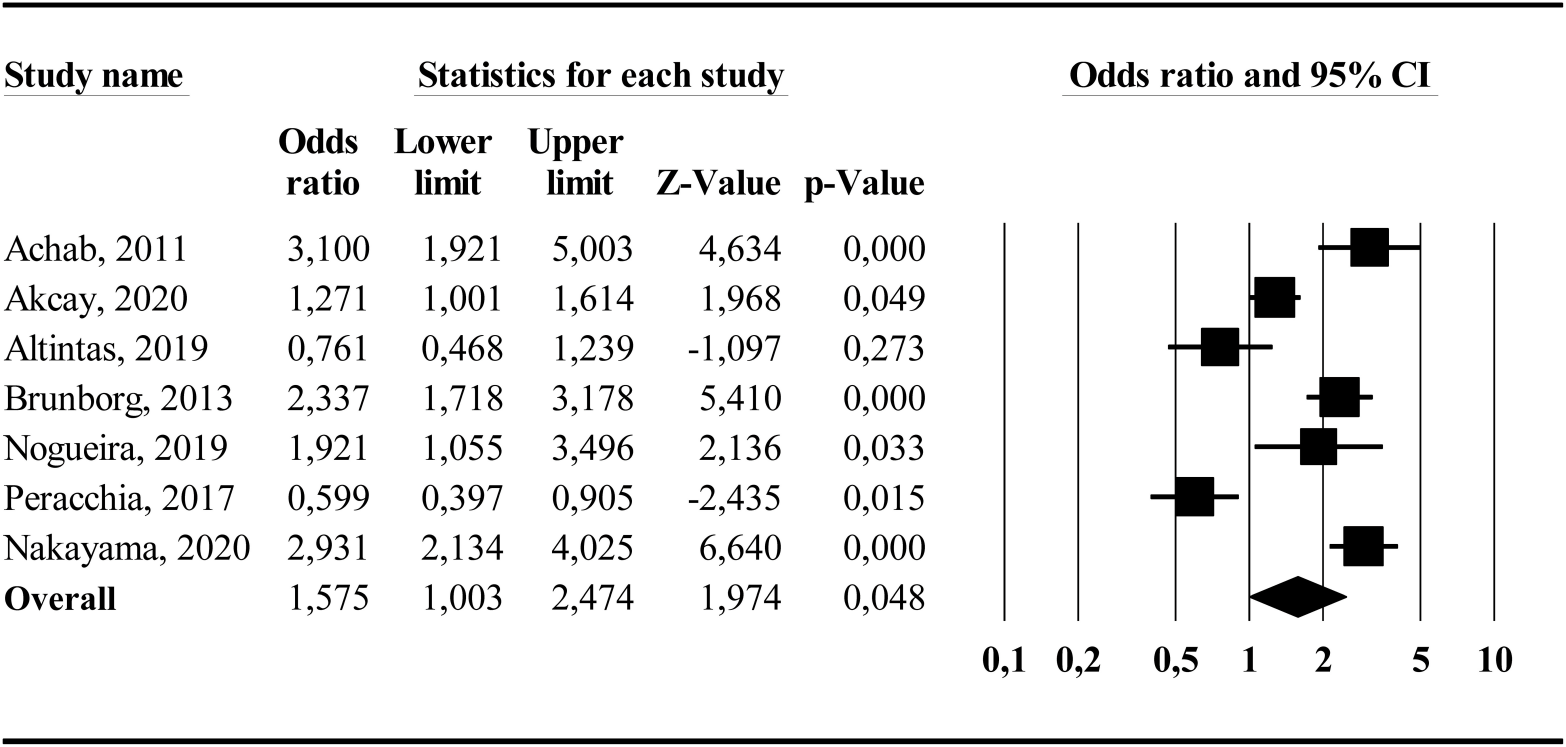
Forest plot showing the association (odds ratio) between problematic gaming and daytime sleepiness.

Figure 4 displays the forest plot for daytime sleepiness. The results from the random-effects model showed a significant overall effect size of *OR* = 1.57, 95% *CI* (1.00, 2.47), indicating that problematic gamers had increased odds of reporting daytime sleepiness compared to non-problematic gamers. The *Q*-statistic was significant (*Q* = 61.75, *df* = 6, *p* < 0.001), and the *I*^2−^statistic showed a high percentage of true between-study variance (*I*^2^ = 90.3%). The subgroup analysis comparing studies targeting adolescent populations (*k* = 4, weighted mean age = 13.3) and studies targeting adult/non-specific age populations (*k* = 3, weighted mean age = 24.1) did not yield any significant differences between subgroups (*Q*_bet_ = 0.09, *df* = 1, *p* = 0.753). The subgroup analysis comparing studies that used a single-item sleep assessment (*k* = 3) and studies using multi-item sleep questionnaires (*k* = 4) showed that the former subgroup had a significant higher overall effect size (*OR* = 2.74, 95% *CI* [1.84, 4.08]) compared to the latter subgroup (*OR* = 1.01, 95% *CI* [0.70, 1.44]), with a *Q*_bet_ = 13.30, *df* = 1, *p* < 0.001. There was still significant heterogeneity within the multi-item sleep questionnaire group (*Q* = 15.12, *df* = 3, *p* < 0.01, *I*^2^ = 80.1%) while there was no significant heterogeneity within the single-item sleep assessment group (*Q* = 1.42, *df* = 2, *p* = 0.491, *I*^2^ = 0.0%). The funnel plot was symmetric, and the trim and fill suggested that there were no missing studies; resulting in no adjustment of the overall effect size. Orwin's fail-safe *N* showed that 12 missing studies with zero effect are needed to reduce the overall effect to the trivial effect criterion (*OR* = 1.2).

#### Sleep Problems

**Figure 5:**
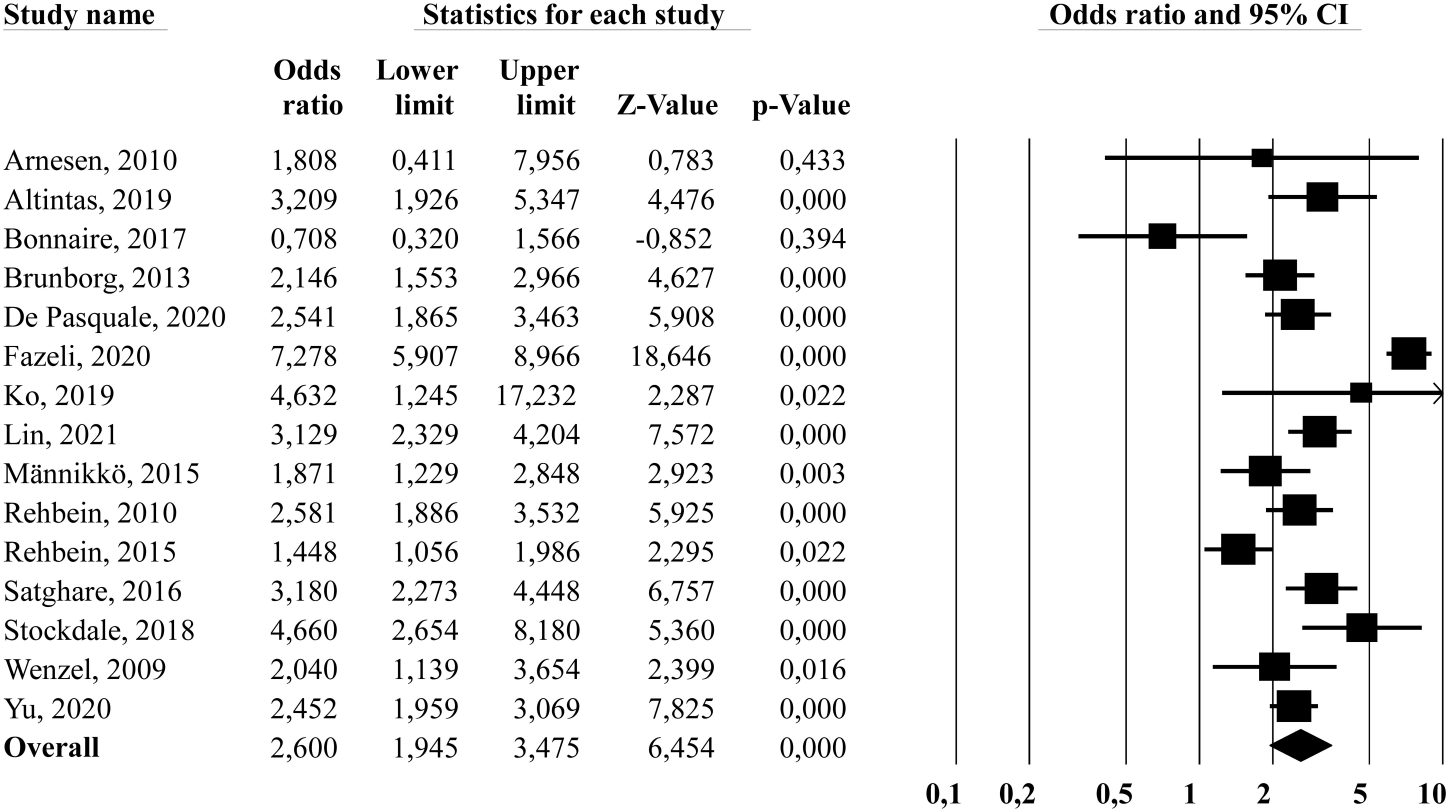
Forest plot showing the association (odds ratio) between problematic gaming and sleep problems.

Figure 5 displays the forest plot for sleep problems. The results from the random-effects model showed a significant overall effect size of *OR* = 2.60, 95% *CI* (1.94, 3.47), indicating that problematic gamers had increased odds of reporting sleep problems compared to non-problematic gamers. The *Q*-statistic was significant (*Q* = 121.62, *df* = 14, *p* < 0.001), indicating significant heterogeneity between the studies. The *I*^2−^statistic showed a high percentage of true between-study variance (*I*^2^ = 88.5%). The subgroup analysis comparing studies targeting adolescent populations (*k* = 8, weighted mean age = 15.0) and studies targeting adult/non-specific age populations (*k* = 7, weighted mean age = 24.6) did not yield any significant differences between subgroups (*Q*_bet_ = 0.61, *df* = 1, *p* = 0.434). There was significant heterogeneity within the adolescent subgroup (*Q* = 115.45, *df* = 7, *p* < 0.001, *I*^2^ = 93.9%) while there was no significant heterogeneity within the adult/non-specific group (*Q* = 6.13, *df* = 6, *p* = 0.409, *I*^2^ = 2.1%). Finally, the subgroup analysis comparing studies that used a single-item sleep measure (*k* = 6) and studies using multi-item sleep questionnaires (*k* = 9) showed a significant difference between groups (*Q*_bet_ = 7.20, *df* = 1, *p* < 0.01), where the multi-item sleep questionnaire subgroup yielded a higher overall effect size (*OR* = 3.47, 95% *CI* [2.51, 4.81]) compared to the single-item sleep measure subgroup (*OR* = 1.77, 95% *CI* [1.23, 2.65]). There was significant heterogeneity within both the multi-item sleep questionnaire group (*Q* = 62.92, *df* = 8, *p* < 0.001, *I*^2^ = 87.3%) and the single-item sleep assessment group (*Q* = 13.05, *df* = 5, *p* = 0.02, *I*^2^ = 61.7%). Concerning publication bias, the funnel plot showed a somewhat asymmetrical distribution. The trim and fill suggested that one study was missing, resulting in an adjustment to *OR* = 2.81, 95% *CI* (2.11, 3.76). The Orwin's fail-safe *N* showed that 76 studies with zero effect are needed to bring the overall effect down to the trivial effect criterion (*OR* = 1.2).

## Discussion

The aim of this meta-analytic review was to determine the associations between problematic gaming and sleep. A total of 33 studies, including 51,430 participants across 26 countries were synthesized quantitatively. Overall, the results indicated that problematic gaming was significantly associated with shorter sleep duration (*g* = −0.238/raw mean difference = −20.8 min) and increased likelihood of reporting poorer sleep quality (*OR* = 2.02), daytime sleepiness (*OR* = 1.57) and sleep problems (*OR* = 2.60). Also, the systematic review suggests that problematic gaming is positively associated with delayed sleep phase disorder, eveningness chronotype, sleep deprivation, later bed- and wake-up times, and nocturnal awakenings. Most studies included in the review and meta-analyses were cross-sectional which precludes conclusions regarding directionality/causality.

### Main Findings

All of the meta-analyzed sleep outcomes yielded significant overall effects. Following the classifications of Cohen (50) and Ferguson (51), the overall effects of *g* = −0.238 and *ORs* ranging from 1.57 to 2.60 could all be considered small. The magnitude of *OR*s is, however, difficult to interpret as the interpretation is dependent on the prevalence rates of the outcome (82, 83). Small effect sizes could still be relevant if the outcome variable is important (theoretically or practically) and when the outcome has high prevalence rates (84, 85). The outcome in this case (i.e., sleep problems) could be argued to be of importance due to the range of adverse consequences it involves and its high and increasing prevalence rates (21). Identifying possible determinants of sleep problems (i.e., problematic gaming) is as such of great societal importance, even if such determinants are rather weakly associated with sleep problems. Further, for the finding regarding sleep duration, one could argue that an average nightly sleep reduction of 20.8 min is clinically meaningful, as having a chronic sleep reduction of that size may have adverse consequences and is likely to be experienced as a problem by the individual (86).

The current findings are comparable to the findings in three recent meta-analyses which found significant associations between internet addiction and sleep duration (Cohens *d* = −0.24) and sleep problems (*OR* = 2.20) (87), problematic smartphone use and poor sleep quality (*OR* = 2.60) (88), and excessive general technology use and shorter sleep duration (Cohen's *d* = −0.25) (89). The latter study also found a positive significant relationship between excessive general technology use and sleep problems. This relationship yielded, however, a smaller effect size (*OR* = 1.33) compared to the effect size between problematic gaming and sleep problems observed in the current study (*OR* = 2.60). As Mei et al. (89) included a variety of different media type use in their synthesis (including general PC use, cell phone, MP3 player, tablet, and TV), this discrepancy in effect sizes might be explained by video games' inherent interactive features which may make them more arousing compared to more “passive” media types (e.g., watching TV) (90–92).

### Subgroup Analyses

There was significant heterogeneity in all meta-analyses. Subgroup analyses yielded no significant group differences regarding sleep quality. The lack of significant difference may, however, be a result of low power in the analyses due to the low number of studies included in each subgroup. A significant difference was found in the overall effect between studies targeting adult and adolescent populations on sleep duration where problematic gaming among adolescents was associated with shorter sleep duration than problematic gaming among adults. Given the different mean ages in the subgroups (weighted mean = 23.7 and 15.2 years) this finding could reflect that younger age is associated with shorter sleep duration among problematic gamers. This might be explained by the literature suggesting that adolescents are more prone to have a delayed sleep schedule compared to adults in general (93–95). A combination of a predisposition for delayed sleep phase together with untimely late-night gaming, which may further delay the sleep schedule, might result in a more severe sleep deficit for adolescents compared to adults (96). Alternatively, as the adult samples in the current subgroup analysis comprised college/university students and young adults, this finding might also be related to the fact that this group typically have more morning flexibility (e.g., freedom in choosing which classes to attend and not) compared to primary/high school students which typically have a more predetermined and mandatory schedule and thus fewer opportunities to compensate for delayed bedtimes with later wake-up times (97).

The second subgroup analysis found that for daytime sleepiness and sleep problems, studies using standardized multi-item sleep questionnaires (e.g., PSQI/ESS) yielded statistically different effect sizes compared to studies using a single-item/own-made sleep measure. These variations in effect sizes may be attributed to the fact that standardized multiple-item measurements have more stable and robust psychometric properties (i.e., have less measuring error) compared to single-item measurements (98). This has been demonstrated empirically in a study that found that a single-item sleep disturbance measurement yielded relatively low sensitivity (i.e., ability to correctly identify sleep problems) compared to a multi-item sleep disturbance scale; suggesting that single-item sleep measurements are less able to correctly discriminate true from false positives (99). Followingly, the effect sizes derived from studies implementing single-item/own-made sleep measurements may be less valid. Thus, the overall effects on daytime sleepiness and sleep problems should be interpreted with this methodological issue in mind.

### Publication Bias

The results show that all meta-analyses, except daytime sleepiness, seemed to be somewhat influenced by publication bias according to the funnel plots and trim and fill procedure. This suggests that, for these syntheses, smaller studies with larger effect sizes may be overrepresented (38, 100). The largest bias was found for sleep duration, hence the finding regarding this sleep parameter should be interpreted with some caution. For sleep quality and sleep problems, the overall effects were only slightly adjusted by the trim and fill, suggesting that the impact of the publication bias on the overall effect was rather modest.

### Limitations of the Included Studies

A limitation of the included studies is that most were cross-sectional. The lack of longitudinal studies makes it impossible to draw inferences on the directionality between problematic gaming and sleep or to speculate about causality. Moreover, only 13 of the 34 included studies adjusted their estimates for confounding factors (see Table 3). Importantly, all except for one study assessed sleep based upon participants' self-report, potentially introducing response bias such as socially desirable responding and the common method bias.

No study used polysomnography, thus there is a paucity of knowledge on the distribution of sleep stages in problematic gamers. Most of the sleep-related instruments used in research, like the Bergen Insomnia Scale (101), Epworth Sleepiness Scale (102), and the PSQI (103), have been validated against polysomnography in previous studies. In terms of instruments used for the assessment of problematic gaming, these seem to a very limited extent to have been validated against clinical or neurobiological data in previous research (104, 105). Although some of the instruments used for assessing sleep and problematic gaming have been validated against objective or clinical data, it is striking that only a few of the studies included in the present meta-analysis employed such data/assessment (41, 77). Further, most of the studies fail to provide a detailed account regarding the nature of the gaming behavior and sleep problems. For instance, none of the studies registered the time of day the gaming took place. Also, few studies differentiated between sleep data from weekdays and weekends/holidays. Further, few studies registered data on the specific game genres that were played. Consequently, little is known whether some game types are stronger associated with sleep impairments than others. In addition, none of the studies registered whether gaming before sleep was motivated by relaxation or excitement, which would affect arousal and consequently sleep latency. Of the 34 included studies, only two used random population sampling (56, 79), while the remaining used convenience sampling. Moreover, the samples were primarily western-European adolescents. Consequently, the findings might not be generalizable beyond these populations. Lastly, only 10 of the 34 studies were large with more than 1,000 participants, which may put restrictions on the analytic power and the precision of their estimates. Taken together, the abovementioned issues suggest that the available literature has some serious limitations which should be addressed in future studies.

### Limitations and Strengths of the Current Review

A limitation of this review is the variability in study populations and spread of operationalizations, measurement tools, and cutoff values to indicate both problematic gaming and sleep problems; suggesting that the current review may have some limitations in terms of comparability between the included studies. This issue can be highlighted by the high levels of heterogeneity found in all the current meta-analyses. In an effort to reduce the heterogeneity, the review could have implemented more stringent inclusion criteria such as only including studies that conceptualized problematic gaming using the four “core addiction” criteria [i.e., conflict, withdrawal, relapse, and problems; cf., (5)] or only included studies adapting the IGD/GD conceptualization. This would, however, further reduce the somewhat restricted pool of studies on the topic and result in insufficient analytic power. Another limitation is the fact that the subgroups of problematic gamers were collapsed in the meta-analyses, although it is conceivable that problematic gamers with more severe symptoms experience more problems with sleep than those with less severe symptoms. Thus, collapsing subgroups may have downplayed the severity of sleep problems among the most problematic gamers. Furthermore, there are also some potential limitations in the present search strategy and inclusion criteria which could cause bias to the synthesis. While the current inclusion criteria did not exclude non-journal manuscripts, the current search strategy makes it likely that scientific work not published in scientific journals could have been overlooked – resulting in possible selection bias. To reduce the risk of bias, the search strategy could have been extended to include gray literature databases (e.g., *ProQuest Dissertations & Theses, OpenGrey*) and would further have benefited from being validated by a librarian and/or through peer review. Moreover, there might be language bias to the synthesis as the current inclusion criteria excluded all manuscripts that were not in a European language, especially considering the high attention surrounding problematic gaming in Southeast Asian countries (14).

Despite these abovementioned limitations, a strength of the current review is the implementation of two independent reviewers in the screening, data extraction, and study quality assessment processes; reducing the risk of bias and human error and consequently increasing the reliability, reproducibility, and internal validity of the synthesis. Moreover, the current study provides the most comprehensive systematic review on the topic to date and is the first study to quantify the relationship between problematic gaming and multiple sleep-related outcomes using a meta-analytic approach.

### Implications for Further Research

This review highlights several future research avenues. The field is in need of more longitudinal studies in order to assess the directionality of the gaming-sleep problems relationship. Moreover, more knowledge is needed on the possible causal pathways involved regarding the relationship between problematic gaming, sleep, and associated outcomes. For instance, the physical, psychological, and functional impairments associated with problematic gaming may reflect an indirect effect between problematic gaming and sleep interference rather than there being a direct causal effect between problematic gaming and negative outcomes. Followingly, future research would benefit from registering more detailed data, such as time of day of gaming, type of game played, sleep both during weekdays and weekends, and specify the nature of the sleep problem as this could provide a better understanding of the mechanisms underlying the association between problematic gaming and sleep. Furthermore, this systematic review and meta-analysis note a potential methodical issue regarding the use of single-item sleep measurement which could yield unprecise estimates. Further research would benefit from assessing sleep parameters using standardized and validated questionnaires as these have less measurement error and would make for better comparisons as well as providing more information on the severity of sleep problems. Also, rather than retrospective questionnaires, further studies would benefit from using sleep diaries as it provides a more detailed picture on the day-to-day relationship between gaming and sleep (e.g., mini-longitudinal design), providing data less prone to recall bias. Moreover, further research would benefit by measuring sleep objectively using polysomnography or actigraphy and to employ clinical screening tools for sleep-related disorders in order to advance the literature. Also, the current evidence base is in need of more nationally representative, and cross-cultural studies, as the currently available data are encumbered with limitations in terms of generalizability.

## Conclusion

The current review contributes to the debate on problematic gaming by offering the first quantitative systematic review on problematic gaming and sleep. Overall, the current synthesis suggests that problematic gaming is associated with sleep impairments, confirming that problematic gaming is related to adverse outcomes. The field is, however, currently in urgent need of more high-quality studies and longitudinal investigations. Insight into sleep's role in problematic gaming behavior is important as it could contribute to a better understanding of the etiology of both problematic gaming and sleep problems, which is important for effective assessments, treatments, and health promotion strategies.

## Data Availability Statement

The raw data supporting the conclusions of this article will be made available by the authors, without undue reservation.

## Author Contributions

JK, EE, and SP conceived and designed the study. JK conducted the literature search and wrote the first draft of the manuscript. JK and EE conducted the data extraction and quality assessment. Statistical analyses were conducted by JK under the supervision of SP. All authors contributed to and have approved the final manuscript.

## Conflict of Interest

The authors declare that the research was conducted in the absence of any commercial or financial relationships that could be construed as a potential conflict of interest.

## References

[1] Interactive Software Federation of Europe. Gametrack Survey 2018. (2019). Available online at: https://www.isfe.eu/wp-content/uploads/2019/08/ISFE-Key-Facts-Brochure-FINAL.pdf (accessed March 2, 2021).

[2] Granic I Lobel A Engels RC. The benefits of playing video games. Am Psychol. (2014) 69:66–78. 10.1037/a0034857 24295515

[3] Adachi PJC Willoughby T. More than just fun and games: the longitudinal relationships between strategic video games, self-reported problem solving skills, academic grades. J Youth Adolesc. (2013) 42:1041–52. 10.1007/s10964-013-9913-9 23344653

[4] Kuss DJ Griffiths MD. Internet gaming addiction: a systematic review of empirical research. Int J Ment Health Addict. (2012) 10:278–96. 10.1007/s11469-011-9318-5

[5] Männikkö N Ruotsalainen H Miettunen J Pontes HM Kääriäinen M. Problematic gaming behaviour and health-related outcomes: a systematic review and meta-analysis. J Health Psychol. (2017) 25:67–81. 10.1177/1359105317740414 29192524

[6] Gentile DA Choo H Liau A Sim T Li D Fung D. Pathological video game use among youths: a two-year longitudinal study. Pediatrics. (2011) 127:e319. 10.1542/peds.2010-1353 21242221

[7] Krossbakken E Pallesen S Mentzoni RA King DL Molde H Finserås TR. A cross-lagged study of developmental trajectories of video game engagement, addiction, mental health. Front Psychol. (2018) 9:2239. 10.3389/fpsyg.2018.02239 30519203 PMC6258776

[8] Kim NR Hwang SS-H Choi J-S Kim D-J Demetrovics Z Király O. Characteristics and psychiatric symptoms of internet gaming disorder among adults using self-reported dsm-5 criteria. Psychiatry Invest. (2016) 13:58–66. 10.4306/pi.2016.13.1.58 26766947 PMC4701686

[9] Brunborg GS Mentzoni RA Frøyland LR. Is video gaming, or video game addiction, associated with depression, academic achievement, heavy episodic drinking, or conduct problems? J Behav Addict. (2014) 3:27–32. 10.1556/JBA.3.2014.002 25215212 PMC4117274

[10] Mentzoni R Brunborg G Molde H Myrseth H Skouverøe K Hetland J. Problematic video game use: estimated prevalence and associations with mental and physical health. Cyberpsychol Behav Soc Netw. (2011) 14:591–6. 10.1089/cyber.2010.0260 21342010

[11] Lemmens JS Valkenburg PM Peter J. Development and validation of a game addiction scale for adolescents. Media Psychol. (2009) 12:77–95. 10.1080/15213260802669458

[12] American Psychological Association. Diagnostic and Statistical Manual of Mental Disorders. Washington, DC: American Psychiatric Publishing (2013). 10.1176/appi.books.9780890425596

[13] World Health Organization. International Classification of Diseases for Mortality and Morbidity Statistics. Geneva: WHO (2018).

[14] Griffiths M Király O Pontes H Demetrovics Z. An overview of problematic gaming. In: Aboujaoude E Starcevic V, editors. Mental Health in the Digital Age: Grave Dangers, Great Promise. Oxford: Oxford University Press (2015). p. 27–45. 10.1093/med/9780199380183.003.0002

[15] Fam JY. Prevalence of internet gaming disorder in adolescents: a meta-analysis across three decades. Scand J Psychol. (2018) 59:524–31. 10.1111/sjop.12459 30004118

[16] Stevens MW Dorstyn D Delfabbro PH King DL. Global prevalence of gaming disorder: a systematic review and meta-analysis. Aust N Z J Psychiatry. (2020). 10.1177/0004867420962851. [Epub ahead of print]. 33028074

[17] Mihara S Higuchi S. Cross-sectional and longitudinal epidemiological studies of internet gaming disorder: a systematic review of the literature. Psychiatry Clin Neurosci. (2017) 71:425–44. 10.1111/pcn.12532 28436212

[18] Van Rooij AJ Ferguson CJ Carras MC Kardefelt-Winther D Shi J Aarseth E. A weak scientific basis for gaming disorder: let us err on the side of caution. J Behav Addict. (2018) 7:1. 10.1556/2006.7.2018.19 29529886 PMC6035022

[19] Aarseth E Bean AM Boonen H Colder Carras M Coulson M Das D. Scholars' open debate paper on the world health organization ICD-11 gaming disorder proposal. J Behav Addict. (2017) 6:267–70. 10.1556/2006.5.2016.088 28033714 PMC5700734

[20] Carras MC Kardefelt-Winther D. When addiction symptoms and life problems diverge: a latent class analysis of problematic gaming in a representative multinational sample of european adolescents. Eur Child Adolesc Psychiatry. (2018) 27:513–25. 10.1007/s00787-018-1108-1 29368254 PMC5895528

[21] Pallesen S Sivertsen B Nordhus IH Bjorvatn B. A 10-year trend of insomnia prevalence in the adult norwegian population. Sleep Med. (2014) 15:173–9. 10.1016/j.sleep.2013.10.009 24382513

[22] Lam LT. Internet gaming addiction, problematic use of the internet, and sleep problems: a systematic review. Curr Psychiatry Rep. (2014) 16:444. 10.1007/s11920-014-0444-1 24619594

[23] ^*^ Hawi NS Samaha M Griffiths MD. Internet gaming disorder in lebanon: relationships with age, sleep habits, academic achievement. J Behav Addict. (2018) 7:70–8. 10.1556/2006.7.2018.16 29486571 PMC6035028

[24] ^*^ Altintas E Karaca Y Hullaert T Tassi P. Sleep quality and video game playing: effect of intensity of video game playing and mental health. Psychiatry Res. (2019) 273:487–92. 10.1016/j.psychres.2019.01.030 30685731

[25] Cain N Gradisar M. Electronic media use and sleep in school-aged children and adolescents: a review. Sleep Med. (2010) 11:735–42. 10.1016/j.sleep.2010.02.006 20673649

[26] Van den Bulck J. Is television bad for your health? Behavior and body image of the adolescent “Couch Potato”. J Youth Adolesc. (2000) 29:273–88. 10.1023/A:1005102523848

[27] Exelmans L Van den Bulck J. Bedtime, shuteye time and electronic media: sleep displacement is a two-step process. J Sleep Res. (2017) 26:364–70. 10.1111/jsr.12510 28271575

[28] Lockley SW Foster RG. Sleep: A Very Short Introduction. New York, NY: Oxford University Press (2012). 10.1093/actrade/9780199587858.001.0001

[29] Dijk D-J Lockley SW. Invited review: integration of human sleep-wake regulation and circadian rhythmicity. J Appl Physiol. (2002) 92:852–62. 10.1152/japplphysiol.00924.2001 11796701

[30] Dijk D-J Archer SN. Light, sleep, and circadian rhythms: together again. PLoS Biol. (2009) 7:e1000145. 10.1371/journal.pbio.1000145 19547745 PMC2691600

[31] Thomée S Dellve L Härenstam A Hagberg M. Perceived connections between information and communication technology use and mental symptoms among young adults-a qualitative study. BMC Public Health. (2010) 10:66. 10.1186/1471-2458-10-66 20152023 PMC2836296

[32] Loughran SP Wood AW Barton JM Croft RJ Thompson B Stough C. The effect of electromagnetic fields emitted by mobile phones on human sleep. Neuroreport. (2005) 16:1973–6. 10.1097/01.wnr.0000186593.79705.3c 16272890

[33] Lowden A Åkerstedt T Ingre M Wiholm C Hillert L. Kuster N, et al. Sleep after mobile phone exposure in subjects with mobile phone-related symptoms. Bioelectromagnetics. (2011) 32:4. 10.1002/bem.20609 20857453

[34] Karasek M Woldanska-Okonska M Czernicki J Zylinska K Swietoslawski J. Chronic exposure to 2.9 mt, 40 hz magnetic field reduces melatonin concentrations in humans. J Pineal Res. (1998) 25:240–4. 10.1111/j.1600-079X.1998.tb00393.x 9885993

[35] Baglioni C Battagliese G Feige B Spiegelhalder K Nissen C Voderholzer U. Insomnia as a predictor of depression: a meta-analytic evaluation of longitudinal epidemiological studies. J Affect Disord. (2011) 135:10–9. 10.1016/j.jad.2011.01.011 21300408

[36] Moher D Liberati A Tetzlaff J Altman DG Prisma Group. Preferred reporting items for systematic reviews and meta-analyses: the prisma statement. PLoS Med. (2009) 6:e1000097. 10.1371/journal.pmed.1000097 19621072 PMC2707599

[37] Page MJ McKenzie JE Bossuyt PM Boutron I Hoffmann TC Mulrow CD. The PRISMA 2020 statement: an updated guideline for reporting systematic reviews. BMJ. (2021) 372:n71. 10.1136/bmj.n71 33782057 PMC8005924

[38] Borenstein M Hedges LV Higgins JP Rothstein HR. Introduction to Meta-Analysis. Chichester: John Wiley and Sons, Ltd (2009). 10.1002/9780470743386

[39] Giang HTN Ahmed AM Fala RY Khattab MM Othman MHA Abdelrahman SAM. Methodological steps used by authors of systematic reviews and meta-analyses of clinical trials: a cross-sectional study. BMC Med Res Methodol. (2019) 19:164. 10.1186/s12874-019-0780-2 31349805 PMC6659247

[40] ^*^ Lin CY Potenza MN Brostrom A Pakpour AH. Internet gaming disorder, psychological distress, and insomnia in adolescent students and their siblings: an actor-partner interdependence model approach. Addict Behav Rep. (2021) 13:100332. 10.1016/j.abrep.2020.100332 33437860 PMC7786042

[41] ^*^ Ko C-H Lin H-C Lin P-C Yen J-Y. Validity, functional impairment and complications related to internet gaming disorder in the dsm-5 and gaming disorder in the icd-11. Aust N Zeal J Psychiatry. (2019) 54:707–8. 10.1177/0004867419881499 31631668

[42] Wells G Shea B O'Connell D Peterson J Welch V Losos M. The Newcastle-Ottawa scale (NOS) for Assessing the Quality of Nonrandomised Studies in Meta-Analyses. (2000). Available online at: http://www.ohri.ca/programs/clinical_epidemiology/oxford.asp (accessed May 12, 2021).

[43] Modesti PA Reboldi G Cappuccio FP Agyemang C Remuzzi G Rapi S. Panethnic differences in blood pressure in europe: a systematic review and meta-analysis. PLoS ONE. (2016) 11:e0147601. 10.1371/journal.pone.0147601 26808317 PMC4725677

[44] King DL Haagsma MC Delfabbro PH Gradisar M Griffiths MD. Toward a consensus definition of pathological video-gaming: a systematic review of psychometric assessment tools. Clin Psychol Rev. (2013) 33:331–42. 10.1016/j.cpr.2013.01.002 23396015

[45] Hale L Guan S. Screen time and sleep among school-aged children and adolescents: a systematic literature review. Sleep Med Rev. (2015) 21:50–8. 10.1016/j.smrv.2014.07.007 25193149 PMC4437561

[46] Higgins JP Thompson SG Deeks JJ Altman DG. Measuring inconsistency in meta-analyses. BMJ. (2003) 327:557–60. 10.1136/bmj.327.7414.557 12958120 PMC192859

[47] Orwin RG. A fail-safe n for effect size in meta-analysis. J Educ Stat. (1983) 8:157–9. 10.2307/1164923

[48] Duval S Tweedie R. A nonparametric “trim and fill” method of accounting for publication bias in meta-analysis. J Am Stat Assoc. (2000) 95:89–98. 10.1080/01621459.2000.10473905

[49] Duval S Tweedie R. Trim and fill: a simple funnel-plot–based method of testing and adjusting for publication bias in meta-analysis. Biometrics. (2000) 56:455–63. 10.1111/j.0006-341X.2000.00455.x 10877304

[50] Cohen J. Statistical Power Analysis for the Behavioral Sciences. Hillsdale, NJ: Erlbaum (1988).

[51] Ferguson CJ. An effect size primer: a guide for clinicians and researchers. In: Kazdin A, editor. Methodological Issues and Strategies in Clinical Research. 4th ed. American Psychological Association (2016). p. 301–10. 10.1037/14805-020

[52] ^*^ Achab S Nicolier M Mauny F Monnin J Trojak B Vandel P. Massively multiplayer online role-playing games: comparing characteristics of addict vs non-addict online recruited gamers in a french adult population. BMC Psychiatry. (2011) 11:144. 10.1186/1471-244X-11-144 21871089 PMC3176476

[53] ^*^ Akçay D Akçay BD. The effect of computer game playing habits of university students on their sleep states. Perspect Psychiatr Care. (2020) 12:1–7. 10.1111/ppc.12497 32163182

[54] ^*^ Al Asqah MI Al Orainey AI Shukr MA Al Oraini HM Al Turki YA. The prevalence of internet gaming disorder among medical students at king saud university, riyadh, saudi arabia. A cross-sectional study. Saudi Med J. (2020) 41:1359–63. 10.15537/smj.2020.12.05584 33294895 PMC7841580

[55] ^*^ Al Gammal MA Ali Elsheikh MM Abozahra AA. Internet addiction and internet gaming disorder and associated insomnia among a sample of Al-Azhar university students, clinical study. Egypt J Hospital Med. (2019) 77:5718–26. 10.21608/ejhm.2019.63227

[56] ^*^ Arnesen AA. Video game addiction among young adults in Norway: prevalence and health (Master thesis). Bergen: University of Bergen (2010).

[57] ^*^ Bonnaire C Phan O. [Negative perceptions of the risks associated with gaming in young adolescents: an exploratory study to help thinking about a prevention program]. Arch Pediatrie. (2017) 24:607–17. 10.1016/j.arcped.2017.04.006 28595830

[58] ^*^ Brunborg GS Mentzoni RA Melkevik OR Torsheim T Samdal O Hetland J. Gaming addiction, gaming engagement, and psychological health complaints among norwegian adolescents. Media Psychol. (2013) 16:115–28. 10.1080/15213269.2012.756374

[59] ^*^ De Pasquale C Sciacca F Martinelli V Chiappedi M Dinaro C Hichy Z. Relationship of internet gaming disorder with psychopathology and social adaptation in italian young adults. Int J Environ Res Public Health. (2020) 17:8201. 10.3390/ijerph17218201 33172015 PMC7664226

[60] ^*^ Fazeli S Mohammadi Zeidi I Lin C-Y Namdar P Griffiths MD. Depression, anxiety, and stress mediate the associations between internet gaming disorder, insomnia, and quality of life during the Covid-19 outbreak. Addict Behav Rep. (2020) 12:100307. 10.1016/j.abrep.2020.100307 33110934 PMC7581367

[61] ^*^ Fernandes B Biswas UN Tan-Mansukhani R Vallejo A Essau CA. The impact of covid-19 lockdown on internet use and escapism in adolescents. Rev Psicol Clin Ninos Adolesc. (2020) 7:59–65. 10.21134/rpcna.2020.mon.2056

[62] ^*^ Gonzalez-Valero G Luis Ubago-Jimenez J Andres Ramirez-Graniz I Puertas-Molero P. Relationship between the use of video games and physical-healthy, psychosocial and academic indicators in primary schoolchildren. J Hum Sport Exerc. (2020) 15:S336–44. 10.14198/jhse.2020.15.Proc2.25

[63] ^*^ Kim N Hughes TL Park CG Quinn L Kong ID. Resting-state peripheral catecholamine and anxiety levels in korean male adolescents with internet game addiction. Cyberpsychol Behav Soc Netw. (2016) 19:202–8. 10.1089/cyber.2015.0411 26849530 PMC4799709

[64] ^*^ King DL Delfabbro PH Zwaans T Kaptsis D. Sleep interference effects of pathological electronic media use during adolescence. Int J Mental Health Addict. (2014) 12:21–35. 10.1007/s11469-013-9461-2

[65] ^*^ Lin C-Y Imani V Brostrom A Arestedt K Pakpour AH. Evaluating the psychometric properties of the 7-item persian game addiction scale for iranian adolescents. Front Psychol. (2019) 10:149. 10.3389/fpsyg.2019.00149 30804841 PMC6370725

[66] ^*^ Liu Y Wang Q Jou M Wang B An Y Li Z. Psychometric properties and measurement invariance of the 7-item game addiction scale (GAS) among chinese college students. BMC Psychiatry. (2020) 20:484. 10.1186/s12888-020-02830-7 33008339 PMC7531159

[67] ^*^ Männikkö N Billieux J Kaariainen M. Problematic digital gaming behavior and its relation to the psychological, social and physical health of finnish adolescents and young adults. J Behav Addict. (2015) 4:281–8. 10.1556/2006.4.2015.040 26690623 PMC4712762

[68] ^*^ Männikkö N Ruotsalainen H Tolvanen A Kääriäinen M. Problematic gaming is associated with some health-related behaviors among finnish vocational school students. Int J Ment Health Addict. (2019) 18:993–1007. 10.1007/s11469-019-00100-6

[69] ^*^ Nakayama H Matsuzaki T Mihara S Kitayuguchi T Higuchi S. Relationship between problematic gaming and age at the onset of habitual gaming. Pediatrics Int. (2020) 62:1275–81. 10.1111/ped.14290 32379947

[70] ^*^ Nogueira M Faria H Vitorino A Silva FG Neto AS. Addictive video game use: an emerging pediatric problem? Acta Med Port. (2019) 32:183–8. 10.20344/amp.10985 30946788

[71] ^*^ Peracchia S Triberti S Curcio G. Longer the game, better the sleep: intense video game playing is associated to better sleep quality and better daytime functioning. Ann Rev CyberTherapy Telemed. (2017) 15:204–6.

[72] ^*^ Rehbein F Kleimann M Mossle T. Prevalence and risk factors of video game dependency in adolescence: results of a German nationwide survey. Cyberpsychol Behav Soc Netw. (2010) 13:269–77. 10.1089/cyber.2009.0227 20557246

[73] ^*^ Rehbein F Kliem S Baier D Mosle T Petry NM. Prevalence of internet gaming disorder in german adolescents: diagnostic contribution of the nine DSM-5 criteria in a state-wide representative sample. Addiction. (2015) 110:842–51. 10.1111/add.12849 25598040

[74] ^*^ Satghare P Abdin E Vaingankar JA Chua BY Pang S Picco L. Prevalence of sleep problems among those with internet gaming disorder in singapore. Asian J Psychiatry. (2016) 17:188–98.

[75] ^*^ Severo RB Soares JM Affonso JP Giusti DA de Souza Junior AA de Figueiredo VL. Prevalence and risk factors for internet gaming disorder. Braz J Psychiatry. (2020) 42:532–5. 10.1590/1516-4446-2019-0760 32785455 PMC7524423

[76] ^*^ Stockdale L Coyne SM. Video game addiction in emerging adulthood: cross-sectional evidence of pathology in video game addicts as compared to matched healthy controls. J Affect Disord. (2018) 225:265–72. 10.1016/j.jad.2017.08.045 28841491

[77] ^*^ Turel O Romashkin A Morrison KM. Health outcomes of information system use lifestyles among adolescents: videogame addiction, sleep curtailment and cardio-metabolic deficiencies. PLoS ONE. (2016) 11:e0154764. 10.1371/journal.pone.0154764 27149512 PMC4858285

[78] ^*^ Vollmer C Randler C Horzum MB Ayas T. Computer game addiction in adolescents and its relationship to chronotype and personality. SAGE Open. (2014) 4:1–9. 10.1177/2158244013518054

[79] ^*^ Wenzel H Bakken I Johansson A Gotestam K Oren A. Excessive computer game playing among norwegian adults: self-reported consequences of playing and association with mental health problems. Psychol Rep. (2009) 105:1237–47. 10.2466/PR0.105.F.1237-1247 20229923

[80] ^*^ Wong HY Mo HY Potenza MN Chan MNM Lau WM Chui TK. Relationships between severity of internet gaming disorder, severity of problematic social media use, sleep quality and psychological distress. Int J Environ Res Public Health. (2020) 17:1879. 10.3390/ijerph17061879 32183188 PMC7143464

[81] ^*^ Yu Y Yang X Wang S Wang H Chang R Tsamlag L. Serial multiple mediation of the association between internet gaming disorder and suicidal ideation by insomnia and depression in adolescents in shanghai, China. BMC Psychiatry. (2020) 20:460. 10.1186/s12888-020-02870-z 32967648 PMC7510306

[82] Chen H Cohen P Chen S. How big is a big odds ratio? Interpreting the magnitudes of odds ratios in epidemiological studies. Commun Stat Simulat Comput. (2010) 39:860–4. 10.1080/03610911003650383

[83] Nemes S Jonasson JM Genell A Steineck G. Bias in odds ratios by logistic regression modelling and sample size. BMC Med Res Methodol. (2009) 9:56. 10.1186/1471-2288-9-56 19635144 PMC2724427

[84] Breaugh JA. Effect size estimation: factors to consider and mistakes to avoid. J Manag. (2003) 29:79–97. 10.1177/014920630302900106

[85] Prentice DA Miller DT. When small effects are impressive. Psychol Bull. (1992) 112:160–4. 10.1037/0033-2909.112.1.160

[86] Endocrine Society. Losing 30 Minutes of Sleep Per Day May Promote Weight Gain and Adversely Affect Blood Sugar Control. ScienceDaily (2015). Available online at: www.sciencedaily.com/releases/2015/03/150306082541.htm (accessed March 02, 2021).

[87] Alimoradi Z Lin C-Y Broström A Bülow PH Bajalan Z. Internet addiction and sleep problems: a systematic review and meta-analysis. Sleep Med Rev. (2019) 47:51–61. 10.1016/j.smrv.2019.06.004 31336284

[88] Sohn S Rees P Wildridge B Kalk NJ Carter B. Prevalence of problematic smartphone usage and associated mental health outcomes amongst children and young people: a systematic review, meta-analysis and grade of the evidence. BMC Psychiatry. (2019) 19:356. 10.1186/s12888-019-2350-x 31779637 PMC6883663

[89] Mei X Zhou Q Li X Jing P Wang X Hu Z. Sleep problems in excessive technology use among adolescent: a systemic review and meta-analysis. Sleep Sci Pract. (2018) 2:9. 10.1186/s41606-018-0028-9

[90] Dworak M Schierl T Bruns T Strüder HK. Impact of singular excessive computer game and television exposure on sleep patterns and memory performance of school-aged children. Pediatrics. (2007) 120:978. 10.1542/peds.2007-0476 17974734

[91] Gradisar M Wolfson AR Harvey AG Hale L Rosenberg R Czeisler CA. The sleep and technology use of americans: findings from the national sleep foundation's 2011 sleep in america poll. J Clin Sleep Med. (2013) 9:1291–9. 10.5664/jcsm.3272 24340291 PMC3836340

[92] Exelmans L Van den Bulck J. Sleep research: a primer for media scholars. Health Commun. (2019) 34:519–28. 10.1080/10410236.2017.1422100 29323936

[93] Carskadon MA Vieira C Acebo C. Association between puberty and delayed phase preference. Sleep. (1993) 16:258–62. 10.1093/sleep/16.3.258 8506460

[94] Paine S-J Gander PH Travier N. The epidemiology of morningness/eveningness: influence of age, gender, ethnicity, and socioeconomic factors in adults (30-49 years). J Biol Rhythms. (2006) 21:68–76. 10.1177/0748730405283154^1^16461986

[95] Crowley SJ Acebo C Carskadon MA. Sleep, circadian rhythms, and delayed phase in adolescence. Sleep Med. (2007) 8:602–12. 10.1016/j.sleep.2006.12.002 17383934

[96] Bartel KA Gradisar M Williamson P. Protective and risk factors for adolescent sleep: a meta-analytic review. Sleep Med Rev. (2015) 21:72–85. 10.1016/j.smrv.2014.08.002 25444442

[97] Hysing M Harvey AG Bøe T Heradstveit O Vedaa Ø Sivertsen B. Trajectories of sleep problems from adolescence to adulthood. Linking two population-based studies from Norway. Sleep Med. (2020) 75:411–7. 10.1016/j.sleep.2020.08.035 32971382

[98] Diamantopoulos A Sarstedt M Fuchs C Wilczynski P Kaiser S. Guidelines for choosing between multi-item and single-item scales for construct measurement: a predictive validity perspective. J Acad Market Sci. (2012) 40:434–49. 10.1007/s11747-011-0300-3

[99] Lallukka T Dregan A Armstrong D. Comparison of a sleep item from the general health questionnaire-12 with the jenkins sleep questionnaire as measures of sleep disturbance. J Epidemiol. (2011) 21:474–80. 10.2188/jea.JE20110023 21986193 PMC3899464

[100] Song F Hooper L Loke Y. Publication bias: what is it? How do we measure it? How do we avoid it? Open Access J Clin Trials. (2013) 5:71–81. 10.2147/OAJCT.S34419

[101] Pallesen S Bjorvatn B Nordhus IH Sivertsen B Hjørnevik M Morin CM. A new scale for measuring insomnia: the bergen insomnia scale. Percept Mot Skills. (2008) 107:691–706. 10.2466/pms.107.3.691-706 19235401

[102] Chervin RD Aldrich MS Pickett R Christian G. Comparison of the results of the epworth sleepiness scale and the multiple sleep latency test. J Psychosom Res. (1997) 42:145–55. 10.1016/S0022-3999(96)00239-5 9076642

[103] Backhaus J Junghanns K Broocks A Riemann D Hohagen F. Test–retest reliability and validity of the pittsburgh sleep quality index in primary insomnia. J Psychosom Res. (2002) 53:737–40. 10.1016/S0022-3999(02)00330-6 12217446

[104] King DL Chamberlain SR Carragher N Billieux J Stein D Mueller K. Screening and assessment tools for gaming disorder: a comprehensive systematic review. Clin Psychol Rev. (2020) 77:101831. 10.1016/j.cpr.2020.101831 32143109

[105] Kaye LK Kuss DJ Rumpf H-J. Conceptual and methodological considerations of gaming disorder and internet gaming disorder. In: El-Guebaly N Carrà G Galanter M Baldacchino AM, editors. Textbook of Addiction Treatment: International Perspectives. Cham: Springer International Publishing (2021). p. 967–77. 10.1007/978-3-030-36391-8_68

